# The Optimal Number of Oocytes Retrieved From PCOS Patients Receiving IVF to Obtain Associated With Maximum Cumulative Live Birth Rate and Live Birth After Fresh Embryo Transfer

**DOI:** 10.3389/fendo.2022.878214

**Published:** 2022-06-23

**Authors:** Rui Jia, Yuanyuan Liu, Rulan Jiang, Xuli Zhu, Liang Zhou, Peipei Chen, Mingya Cao, Zhiming Zhao

**Affiliations:** Department of Reproductive Medicine, The Second Hospital of Hebei Medical University, Shijiazhuang, China

**Keywords:** cumulative live birth rate, fresh embryo transfer, polycystic ovarian syndrome, number of oocytes retrieved, *in vitro* fertilization-embryo transfer

## Abstract

**Aims:**

This study aims to determine the optimal number of oocytes retrieved so that patients with polycystic ovary syndrome (PCOS) receiving *in vitro* fertilization (IVF) can obtain the best cumulative live birth rate (CLBR) and live birth after fresh embryo transfer.

**Methods:**

This is a retrospective study of 1,419 patients with PCOS who underwent their first IVF cycle at the Second Hospital of Hebei Medical University from January 2014 to December 2021. Multivariable regression analysis was performed to adjust for factors known to independently affect cumulative live birth aspiration. The number of oocytes retrieved to obtain the best cumulative live birth rate was explored through curve fitting and threshold effect analysis. The decision tree method was used to explore the best number of oocytes retrieved to achieve live birth in the shortest time.

**Results:**

(1) The number of oocytes retrieved was found to be an independent protective factor for the cumulative live birth rate (OR = 1.09 (95% CI: 1.06, 1.12)). When the number of oocytes retrieved was less than 15, CLBR increased by 16% with each increase in the number of oocytes retrieved (OR = 1.16 (95% CI: 1.11, 1.22)); and when more than 15, CLBR tended to be stable. (2) Live birth after the first fresh embryo transfer was analyzed through a classification decision tree. For patients younger than 35 years old, those with less than 6 oocytes and those with 7–16 oocytes had a similar proportion of live births with fresh embryo transfer but higher than 16 oocytes (53.7% vs. 53.8% vs. 18.4%). Patients older than 35 years old had a similar proportion of live births with fresh embryo transfer (35.7% vs. 39.0%) to those younger than 35 years old, but the proportion of no live births after using up all embryos was higher than those younger than 35 years old (39.3% vs. 19.2%).

**Conclusions:**

In PCOS patients, high CLBR can be obtained when the number of oocytes retrieved was 15 or more. The number of oocytes retrieved from 7 to 16 could achieve more chance of live birth after fresh embryo transfer.

## Introduction

Assisted reproductive technology (ART), especially *in vitro* fertilization (IVF), has a major advantage in promoting live birth for infertility patients after years of development. Continuously optimizing the implementation process is of great significance for promoting live births of patients undergoing IVF. In the implementation of IVF, the increased number of oocytes retrieved improved pregnancy rates in women by increasing the number of available embryos for transfer (1). The number of oocytes retrieved is an important factor affecting the cumulative live birth rate, and the cumulative live birth rate (CLBR) increases with the increase in the number of oocytes retrieved (2–4). However, increasing the number of oocytes retrieved not only increases the incidence of ovarian hyperstimulation syndrome (OHSS) and other complications but also increases the possibility of whole embryo freezing, which prolongs the treatment cycle of patients. In addition, the outcome of fresh embryo transfer heralds the success of subsequent frozen embryo transfer using blastocysts from the same cohort (5). It is important to find a suitable number of oocytes retrieved to be obtained so that the patient can obtain a live birth in a short time as soon as possible, that is, a live birth in fresh embryo transfer, both for the current transfer cycle and the subsequent freeze-thaw cycle.

Polycystic ovary syndrome (PCOS) is an endocrine disease characterized by hyperandrogenism, oligoovulation or anovulation, and polycystic ovarian changes. It is one of the factors that cause infertility. Different from patients with other infertility factors, patients with PCOS generally have a large number of the antral follicle and high ovarian reactivity (6). They are easy to obtain more oocytes but the increased number of oocytes increases the risk of OHSS and thrombosis (7). Therefore, in the process of ovarian stimulation, the occurrence of complications such as OHSS is avoided by choosing a milder ovarian stimulation protocol and reducing the dose of gonadotropin, which leads to a reduction in the number of oocytes retrieved. Moreover, the quality of oocytes from PCOS patients is often poor, which leads to lower fertilization, cleavage, implantation, and high pregnancy loss rates (8). Therefore, it is necessary to explore the optimal number of oocytes retrieved to maintain the balance between cumulative live birth and efficient live birth in patients with PCOS.

Only a few studies have shown that the optimal CLBR could be achieved when the number of oocytes retrieved in PCOS patients was 10, and there was no significant difference in the live birth rate between fresh embryo transfer (ET) and frozen embryo transfer (FET) when the number of oocytes retrieved in PCOS patients was less than 16 (7, 9), however, how to achieve the optimally remains controversial. How to control the number of oocytes retrieved to allow PCOS patients to obtain live births by fresh ET deserves further exploration.

The aim of this current study is to investigate the CLBR after one IVF cycle including all fresh and subsequent frozen-thaw embryos in PCOS women. In addition, we wanted to investigate the relationship between the number of oocytes retrieved and live birth in a time as short as possible.

## Materials and Methods

### Patient Population

The patients with PCOS who underwent the first IVF cycle with gonadotropin-releasing hormone antagonist (GnRH-ant) protocol in the Second Hospital of Hebei Medical University from January 2014 to December 2021 were included. PCOS was diagnosed according to the 2003 Rotterdam ESHRE/ASRM PCOS Consensus Workshop Group diagnostic criteria, the presence of ≥ 2 criteria (1) oligoanovulatory ovarian dysfunction (OAD), (2) clinical manifestations of androgen excess and/or hyperandrogenism (HA), (3) PCOM: ultrasonography showing polycystic ovarian morphology (PCOM) (unilateral ovary or bilateral ovaries have ≥ 12 ovarian follicles, 2-9 mm in diameter, and/or ovarian volume >10 mL). Based on the combination of symptoms, PCOS phenotypes are divided into the following: phenotypes A: OAD, HA, and PCOM; phenotypes B: OAD and HA without PCOM; phenotypes C: HA and PCOM without OAD; and phenotypes D: OAD and PCOM without HA.

Exclusion criteria included: (1) endocrine abnormalities such as congenital adrenal hyperplasia, Cushing syndrome, and androgen-secreting tumors; (2) abnormal thyroid function and decreased ovarian reserve function; (3) uterine cavity malformation, endometrial lesion, and genital malformation; (4) oocyte donation, oocyte freezing, preimplantation genetic diagnosis, or preimplantation genetic screening; (5) did not obtain a live birth but did not use up all the embryos; and (6) women age >40.

The patients included in the study were divided into low-oocyte, middle-oocyte, and high-oocyte groups according to the number of oocytes obtained for further description and research.

All procedures performed in studies involving human participants were in accordance with the ethical standards of the institutional and/or national research committee and with the 1964 Helsinki declaration and its later amendments or comparable ethical standards. The study protocol was approved by the ethics committee of the Second Hospital of Hebei Medical University (Hebei, China, ID: 2022-R012).

### Ovarian Stimulation and Oocyte Retrieval

Patients adopted the GnRH-ant protocol. Gonadotropin (Gn, Recombinant Human Follitropin Alfa for Injection, MerckSeronoS.p.A, Italy specification: 75 IU) was used for ovarian stimulation starting on the second day of the menstrual cycle. Serum hormone levels and transvaginal ultrasound were used to monitor follicular development and adjust the dosage of Gn. GnRH-ant was added daily after the leading follicle reached a diameter of 14 mm and carried on until the trigger day. Under ultrasound, when the maximum follicular diameter was ≥18 mm or at least 3 follicles with a diameter of ≥17 mm, recombinant human chorionic gonadotropin α (Recombinant Human Chorionic Gonadotropin Alfa for Injection, MerckSeronoS.p.A., Italy, specification: 250 μg) was administered by injection in 250 μg at night on the same day according to the serum hormone level. If the patient had too many follicles under ultrasound monitoring before the trigger, triptorelin acetate (triptorelin, Ferring GmbH, Germany, specifications: 1 ml; 0.1 mg) was administered by injection in 0.1–0.2 mg to prevent OHSS. Oocyte retrieval was conducted by vaginal ultrasound guided from 36 to 37 h after accepting the trigger.

### IVF and Embryo Culture

After oocyte retrieval, a short-term fertilization scheme was used. At 17 h after fertilization, the fertilization was judged by observing the formation of the pronucleus (10, 11). Embryos were assessed for their morphology and cell number 72 h after fertilization. According to the Istanbul Consensus (10) and Vienna Consensus (11), when the blastomere of 7–9 cells was uniform and the fragmentation rate was less than 10%, the embryo was classified as grade 1. The blastomeres of grade 2 embryos were not very regular, with a diameter difference of less than 20%, and the fragmentation rate ranged between 11% and 25%. The cell division of grade 3 embryos was not uniform, with a diameter difference of 20%–50% and a fragmentation rate of more than 25%. Grade 1 embryos are considered high-quality embryos. Available embryos were defined as grade 1–2 embryos. Both transferred and frozen embryos met the criteria of available embryos.

### Luteal Support and Pregnancy Follow-Up

In fresh cycles, 1–2 available cleavage embryos were transferred on the 3rd day after oocyte retrieval, or one blastocyst of grade >3CC was transferred on the 5th day after oocyte retrieval.

In frozen cycles, a hormone replacement protocol was used to transform the endometrium of the patient, with oral administration of progynova (Delpharm Lille SAS, Bayer Leverkusen, Germany, specification: 1 mg), 2–3 mg twice daily, starting on the 3rd day of menstruation. The patients returned to the hospital on the 12th day of menstruation. For patients with an endometrial thickness of ≥8 mm and *E*_2_ of ≥200 pg/ml, vaginal progesterone (Progesterone Sustained-Release Vaginal Gel; MerckSeronoS.p.A, specification: 90 mg) was used for luteal support. On the 3rd day of corpus luteum support, 1–2 available cleavage embryos were transplanted to the patient, or on the 5th day of corpus luteum support, one blastocyst of grade >3CC was transplanted to the patient. The frozen embryos were thawed according to the rapid recovery method of vitrification, and the surviving blastomeres with a thawing percentage of >50 were used for transplantation.

Progesterone was used for luteal support after embryo transfer. Serum β-hCG was detected 14 days after embryo transfer to determine whether it was a biochemical pregnancy. About 4 weeks after embryo transfer, the gestational sac and heart tube beat were observed under transvaginal ultrasound. The gestational sacs were certified as clinical pregnancy features, and the number of gestational sacs was recorded. The follow-up involved miscarriage or birth and ended until the termination of the pregnancy.

### Observation and Statistical Indicators

The patient endpoint was cumulative live birth or use up of all embryos. Clinical pregnancy was defined as having a gestational sac and primitive heart tube beating. Live birth was defined as being born alive. Patients were classified according to pregnancy outcomes: clinical pregnancy after fresh ET, clinical pregnancy after the first transfer using frozen embryo, clinical pregnancy after multiple embryo transfer, and no clinical pregnancy after using up all embryos. And patients were classified according to live birth outcomes: live birth after fresh ET, live birth after the first transfer using frozen embryo, live birth after multiple embryo transfers, and no live birth after using up all embryos. Laboratory outcome indicators were calculated as follows: Fertilization rate: number of fertilizations/number of oocytes. 2PN fertilization rate: number of 2PN/oocyte number. Cleavage rate: number of 2PN cleavages/number of 2PN. High-quality embryo rate: number of high-quality embryos/number of oocytes. Available embryo rate: the number of available embryos/the number of oocytes.

Clinical outcome indicators were calculated as follows. Cumulative pregnancy rate (CPR): number of clinical pregnancy cycles/total number of first oocyte retrieval cycles. CLBR: number of live birth cycles/total number of first oocyte retrieval cycles. Clinical pregnancy rate: the number of clinical pregnancy cycles after the first transfer/total number of cycles. Live birth rate: the number of live birth cycles after the first transfer/total number of cycles. Abortion rate: number of abortion cycles/number of all clinical pregnancy cycles.

### Statistical Analysis

The statistical analysis was performed with SPSS25.0 software and the statistical packages R (The R Foundation; version 3.4.3) and Empower (R) (X&Y solutions, Boston, MA, USA). Measurement data were expressed as mean ± standard deviation (SD). The counting data were expressed in percent.

For normally distributed data, a two-independent sample test was used to compare the means between two groups, and the analysis of variance method was used to compare the means between multiple groups. For nonnormally distributed data, a nonparametric test (Mann–Whitney *U* test) was used to compare the means. The Chi-square test, or Fisher’s exact probability method, is used to compare count data. Univariate logistic regression analyses were performed to analyze various factors affecting clinical outcomes. After adjusting for confounding factors, both smooth curve fitting and multivariate logistic regression analyses were used to observe the relationship between the number of oocytes retrieved and clinical outcomes. The smooth curve fitting and threshold effect value were combined to quantify the effect of the number of oocytes retrieved on clinical outcomes and stratify by age, BMI and type of PCOS. The SPSS classification decision tree adopted the growth method CHAID. The statistically significant *p* was set at <0.05.

## Results

### Patients’ Characteristics

**Figure 1:**
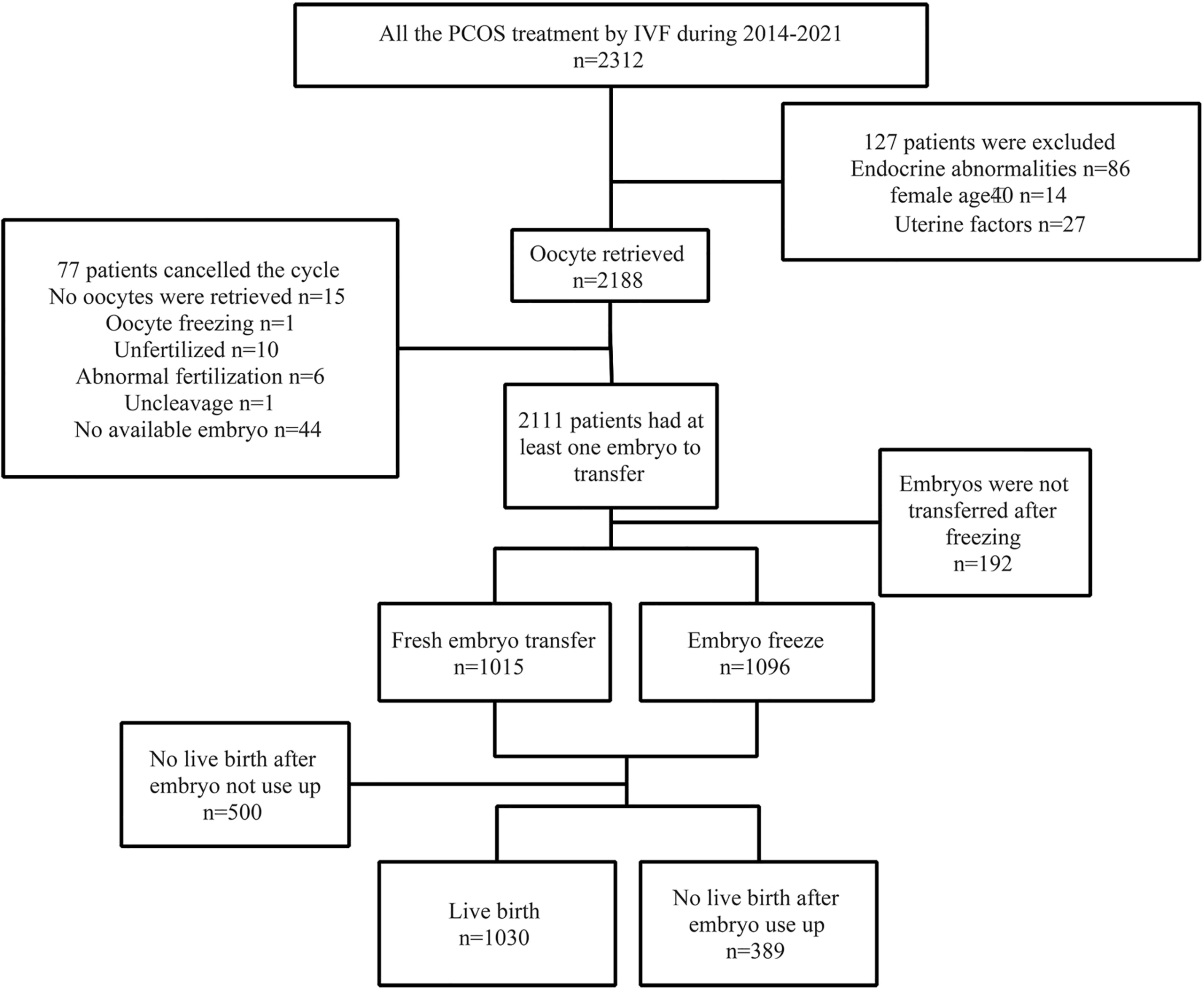
Test flow chart. Patient selection and cycle overview.

After planned exclusions, there are 1,419 PCOS patients with their first IVF treatment eligible for analysis (**Figure 1**). Patients’ characteristics are summarized in **Table 1**. The age, basal follicle-forming hormone (bFSH), dose of Gn, duration of Gn, and the proportion of patients with type B PCOS were significantly higher in the low-oocyte group (1–11) than in the middle-oocyte group (12–17) and high-oocyte group (18–62) (*p* < 0.05). The body mass index (BMI), and total testosterone were significantly higher in the low-oocyte group than in the high-oocyte group (*p* < 0.05). The baseline luteinizing hormone (bLH) was significantly lower in the low-oocyte group than in the middle-oocyte and high-oocyte groups (*p* < 0.05). Patients with fewer than 11 oocytes were more likely to choose fresh ET (90.23%). The patient’s laboratory indicators and clinical outcomes are summarized in **Table 2**. The number of high-quality embryos and the number of available embryos were significantly lower in the low-oocyte group than in the middle-oocyte and high-oocyte groups (*p* < 0.05), but the available embryo rate was significantly lower in the high-oocyte group than in the middle-oocyte and low-oocyte groups (*p* < 0.05), and the high-quality embryo rate was significantly higher in the middle-oocyte group than in the high-oocyte and low-oocyte groups (*p* < 0.05). The clinical pregnancy rate, live birth rate, CPR, and CLBR were significantly higher in the low-oocyte group than in the middle-oocyte and high-oocyte groups (*p* < 0.05), whereas the miscarriage rate was significantly higher in the low-oocyte group than in the middle-oocyte and high-oocyte groups (*p* <0.05).

**Table 1:** Patient/s baseline characteristics.

No. of oocytes	Low (1–11)	Middle (12–17)	High (18–62)	*F*/*χ* ^2^	*p*-value
*N*	471	420	528		
Age (years)	29.22 (3.56)	28.58 (3.52)^*^	28.13 (3.20)^*^	21.834	<0.001
Years of infertility (years)	4.14 (2.65)	4.01 (2.76)	3.75 (2.40)	4.478	0.107
BMI (kg/m^2^)	25.46 (3.83)	25.07 (3.70)	24.74 (3.40)^*^	9.830	0.007
BFSH (mIU/ml)	7.03 (1.87)	6.54 (1.73)^*^	6.36 (1.63)^*^	36.028	<0.001
bE2 (pg/ml)	80.37 (77.16)	74.40 (65.96)	65.48 (53.40)	2.792	0.248
bP (ng/ml)	1.33 (1.34)	1.12 (0.93)	1.21 (1.19)	0.642	0.725
bLH (mIU/ml)	8.53 (6.55)	8.30 (6.09)^*^	9.39 (6.01)^*^	17.044	<0.001
bT (ng/ml)	1.35 (2.93)	1.25 (2.88)	1.01 (1.19)^*^	13.904	<0.001
No. of AFC	19.57 (6.24)	21.73 (5.42)^*^	23.37 (3.97)^*,**^	106.632	<0.001
Dose of Gn (IU)	2,866.04 (1,293.35)	2,443.79 (921.71)^*^	2,372.50 (1,076.41)^*^	52.595	<0.001
Duration of Gn (IU)	12.63 (4.18)	11.73 (2.76)^*^	11.77 (2.94)^*^	12.640	0.002
Types of infertility				5.081	0.079
Primary	309 (65.61%)	257 (61.19%)	360 (68.18%)		
Secondary	162 (34.39%)	163 (38.81%)	168 (31.82%)		
Type of PCOS				91.202	<0.001
A	74 (15.71%)	75 (17.86%)	129 (24.43%)		
B	157 (33.33%)	92 (21.90%)	48 (9.09%)		
C	38 (8.07%)	37 (8.81%)	57 (10.80%)		
D	202 (42.89%)	216 (51.43%)	294 (55.68%)		
Cycle outcome				516.754	<0.001
Fresh embryo transfer	425 (90.23%)	312 (74.29%)	122 (23.11%)		
Frozen embryo transfer	46 (9.77%)	108 (25.71%)	406 (76.89%)		
Number of transferred cycles				16.592	<0.001
1	415 (88.11%)	356 (84.76%)	491 (92.99%)		
2	56 (11.89%)	64 (15.24%)	37 (7.01%)		

^*^p < 0.05 compared with the low group; ^**^p < 0.05 compared with the middle group.

Values are presented as number (%) or mean (SD). BMI, body mass index; bFSH, basal follicle-stimulating hormone; bE2, baseline estradiol; bP, baseline progesterone; bT, baseline testosterone; bLH, baseline luteinizing hormone; AMH, anti-Mullerian hormone; Gn, gonadotropin.

**Table 2:** Patient’s laboratory indicators and clinical outcome.

No. of oocytes	Low (1–11)	Middle (12–17)	High (18–62)	*F*/*χ* ^2^	*p*-value
*N*	471	420	528		
Fertilization rate	83.5%	84.3%	84.0%	5.533	0.063
2PN fertilization rate	61.82%	63.69%	62.03%	1.105	0.575
Cleavage rate	98.87%	98.93%	98.77%	0.629	0.730
High-quality embryo rate	14.20%	14.68%	13.19%	8.492	0.014
Available embryo rate	37.25%	32.63%	27.30%	152.641	<0.001
No. of oocyte	7.58 (2.46)	14.35 (1.70)^*^	24.56 (6.69)^*,**^	1,258.379	<0.001
No. of fertilization	6.34 (2.45)	12.11 (2.68)^*^	20.64(6.76)^*,**^	1,075.068	<0.001
No. of 2PN	4.70 (2.22)	9.14 (2.90)^*^	15.24 (6.02)^*,**^	878.993	<0.001
No. of cleavage	4.64 (2.21)	9.05 (2.88)^*^	15.05 (6.00)^*,**^	871.276	<0.001
No. of available embryo	2.83 (1.47)	4.68 (2.63)^*^	6.70 (3.72)^*,**^	364.865	<0.001
No. of high quality embryo	1.08 (1.43)	2.11 (2.32)^*^	3.24 (3.56)^*,**^	104.923	<0.001
Total number of transferred embryo	2.14 (0.70)	2.27 (0.75)^*^	2.11 (0.53)^*,**^	16.031	<0.001
Clinical pregnancy	312 (66.24%)	336 (80.00%)	468 (88.64%)	74.990	<0.001
Live birth	280 (59.45%)	312 (74.46%)	454 (85.98%)	90.572	<0.001
Miscarriage	32 (10.25%)	24 (7.14%)	14 (2.99%)	17.426	<0.001
Type of abortion				1.395	0.498
Early abortion	21 (65.62%)	12 (50.00%)	8 (57.14%)		
Late abortion	11 (34.38%)	12 (50.00%)	6 (42.86%)		
Cumulative clinical pregnancy	338 (71.76%)	368 (87.62%)	488 (92.42%)	85.055	<0.001
Cumulative live birth	301 (63.91%)	351 (83.57%)	478 (90.53%)	114.501	<0.001
Developmental stage of embryos transferred in live birth					
Cleavage embryo	279 (99.64%)	308 (98.72%)	446 (98.24%)	2.723	0.252
Blastocyst	1 (0.36%)	4 (1.28%)	8 (1.76%)		

^*^p < 0.05 compared with the low group; ^**^p < 0.05 compared with the middle group.

Values are presented as number (%) or mean (SD).

### The Number of Pocytes Retrieved Is an Independent Protective Factor for Pregnancy Outcome

After adjusting for confounding factors (age, BMI, bFSH, bE_2_, dose of Gn, type of PCOS, years of infertility, cycle outcome), multivariate logistic regression analysis showed that the number of oocytes retrieved was an independent protective factor to significantly (*p* < 0.05) affect the clinical pregnancy rate [OR = 1.05 (95% CI: 1.03, 1.08)], live birth rate (OR = 1.05 (95% CI: 1.02, 1.07)), CPR [OR = 1.09 (95% CI: 1.06, 1.12)], and CLBR [OR = 1.09 (95% CI: 1.06, 1.12)] (**Table 3**). For each additional oocyte, the clinical pregnancy rate increased by 5%, the live birth rate increased by 5%, the CPR increased by 9%, and the CLBR increased by 9%. The number of oocytes retrieved is not related to the miscarriage rate.

**Table 3:** Number of oocytes retrieved and clinical outcomes.

	Nonadjusted		Adjusted^a^	
	OR (95% CI)	*p*-value	OR (95% CI)	*p*-value
Cumulative clinical pregnancy	1.10 (1.08, 1.13)	<0.0001	1.09 (1.06, 1.12)	<0.0001
Cumulative live birth	1.11 (1.08, 1.13)	<0.0001	1.09 (1.06, 1.12)	<0.0001
Clinical pregnancy	1.09 (1.07, 1.11)	<0.0001	1.05 (1.03, 1.08)	<0.0001
Live birth	1.09 (1.07, 1.11)	<0.0001	1.05 (1.02, 1.07)	<0.0001
Miscarriage	0.93 (0.90, 0.97)	0.0003	0.98 (0.93,1.02)	0.3143

a Adjusted age, BMI, bFSH, bE_2_, dose of Gn, type of PCOS, years of infertility, and cycle outcome.

**Figure 2:**
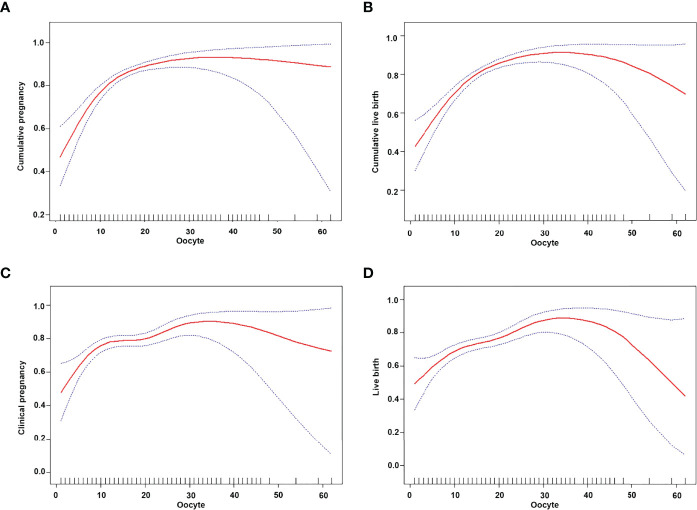
Curve fitting between the number of oocytes retrieved and clinical outcomes. The adjusted smoothed plots between the number of oocytes retrieved with the cumulative pregnancy rate, the cumulative live birth rate, the clinical pregnancy rate, and the live birth rate are based on a two-piece-wise regression model **(A–D)**. The nonlinear relationship between the number of oocytes retrieved and the cumulative pregnancy rate, the cumulative live birth rate, the clinical pregnancy rate, and the live birth rate, respectively. Adjustment factors included age, BMI, bFSH, bE2, dose of Gn, type of PCOS, years of infertility, and cycle outcome. The solid and dashed lines represent the estimated values and their corresponding 95% confidence intervals.

After adjusting for confounding factors such as age, BMI, bFSH, bE_2_, dose of Gn, type of PCOS, years of infertility, and cycle outcome, the smooth curve fitting analysis revealed that the number of oocytes had a curvilinear relationship with the clinical pregnancy rate, live birth rate, CPR, and CLBR (**Figure 2**).

When the number of oocytes was less than 15, the CPR increased by 17%, and CLBR increased by 16% for every increase in the number of retrieved oocytes [OR = 1.17 (95% CI: 1.11, 1.22] and OR = 1.16 [95% CI: 1.11, 1.21)], while the CPR and CLBR plateaued at 15 oocytes (**Table 4**).

**Table 4:** The threshold effect analysis of the number of oocytes retrieved and clinical outcomes.

Cut points	*N*	OR	95% CI	*p*-value
The cumulative pregnancy rate				
<15	695	1.17	(1.11,1.22)	<0.0001
>15	724	1.02	(0.98, 1.06)	0.3825
The cumulative live birth rate				
<15	695	1.16	(1.11,1.21)	<0.0001
>15	724	1.02	(0.98,1.06)	0.2790
The clinical pregnancy rate				
<11	409	1.14	(1.06,1.22)	0.0002
>11, <25	808	1.01	(0.96,1.05)	0.8243
>25	202	0.95	(0.88,1.03)	0.2352
The live birth rate				
<11	409	1.10	(1.02,1.17)	0.0079
>11, <25	808	1.03	(0.98, 1.07)	0.2257
>25	202	0.93	(0.86,0.99)	0.0277

Adjusted age, BMI, bFSH, bE2, dose of Gn, type of PCOS, years of infertility, and cycle outcome.

When the number of oocytes was less than 11, the clinical pregnancy rate and live birth rate increased by 14% and 10%, respectively, for each increase in the number of oocytes [OR = 1.14 (95% CI: 1.06, 1.22) and OR = 1.10 (95% CI: 1.02, 1.17)]. When the number of oocytes was between 11 and 25, the clinical pregnancy rate increased by 1%, and the live birth rate increased by 3% for each increase in the number of oocytes [OR = 1.01 (95% CI: 0.96, 1.05) and OR = 1.03 (95% CI: 0.98, 1.07)]. When the number of oocytes was more than 25, the live birth rate decreased by 7% for each increase in the number of oocytes [OR = 0.93 (95% CI: 0.86, 0.99)], while the clinical pregnancy rate plateaued (**Table 4**).

### Stratified Analysis

**Figure 3:**
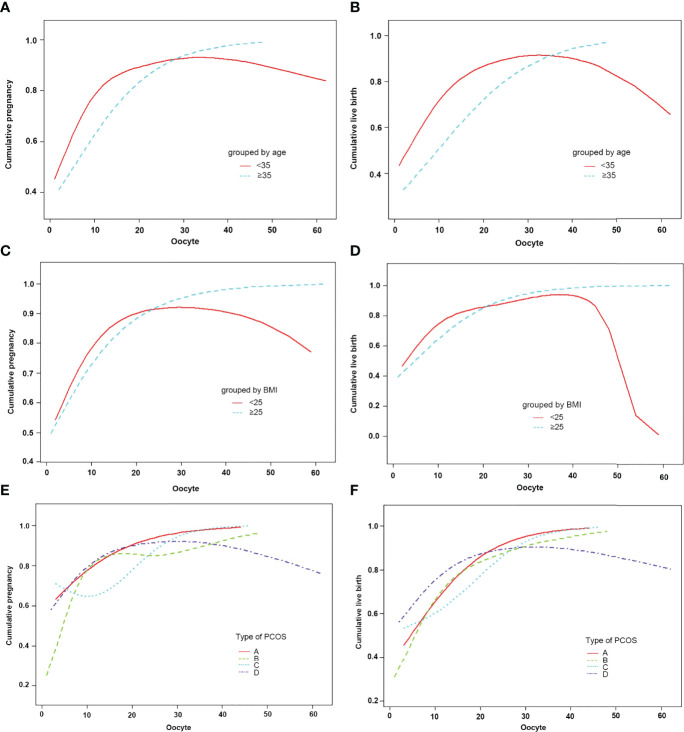
Patients were classified according to age, BMI, type of PCOS stratification, curve fitting between the number of oocytes retrieved, and cumulative clinical outcomes. The adjusted smoothed plots between the number of oocytes retrieved with the cumulative pregnancy rate and the cumulative live birth rate are based on a piecewise regression model **(A–F)**. In **(A, B)**, the red line represents the age less than 35 years old, and the blue line represents the age greater than or equal to 35 years old. In **(C, D)**, the red line represents the BMI of less than 25, and the blue line represents the BMI of greater than or equal to 25. In **(E, F)**, the red line represents the phenotype A, the green line represents the phenotype B, the blue line represents the phenotype C, and the purple line represents the phenotype D. Adjustment factors included age, BMI, bFSH, bE2, dose of Gn, type of PCOS, years of infertility, cycle outcome, and if not stratified.

The subjects were stratified by age. When the age was less than 35 years old, the number of oocytes had a curvilinear relationship with the CPR and CLBR. When the number of oocytes was less than 15, the CPR and CLBR increased by 17% and 16% with each increase in the number of oocytes [OR = 1.17)95% CI: 1.12, 1.23) and OR = 1.16 (95% CI: 1.11, 1.22)]. When there were more than 15, the CPR and CLBR tended to be stable (**Figures 3A, B**; **Table 5**, **6**). When the age was over 35 years old, the number of oocytes obtained had no significant correlation with CPR and CLBR.

Subjects were stratified by BMI. When BMI was less than 25, the number of oocytes showed a curve relationship with the CPR and CLBR. When the number of oocytes was less than 15, CPR and CLBR increased by 18% and 15%, respectively [OR = 1.18 (95% CI: 1.10, 1.26) and OR = 1.15 (95% CI: 1.08, 1.23)]. CPR and CLBR tended to be stable when more than 15 were added (**Figure 3C**; **Table 6**). When BMI was greater than or equal to 25, CPR and CLBR increased by 11% and 12%, respectively, with increasing number of oocytes (OR = 1.11 [95% CI: 1.07, 1.16) and OR = 1.12 (95% CI: 1.08, 1.17)] (**Figure 3D**; **Table 5**).

**Table 5:** Stratified analysis of the number of oocytes retrieved and cumulative clinical outcomes.

	*N*	The cumulative pregnancy rate			The cumulative live birth rate		
		OR	95% CI	*p*-value	OR	95% CI	*p*-value
Age <35	1,335	1.09	(1.06, 1.13)	<0.0001	1.09	(1.06, 1.12)	<0.0001
Age ≥35	84	1.06	(0.96, 1.18)	0.2509	1.08	(0.99, 1.18)	0.0932
BMI <25	725	1.08	(1.03, 1.12)	0.0004	1.06	(1.02, 1.10)	0.002
BMI ≥25	683	1.11	(1.07, 1.16)	<0.0001	1.12	(1.08, 1.17)	<0.0001
Type of PCOS							
A	278	1.11	(1.03, 1.19)	0.005	1.14	(1.07, 1.21)	<0.0001
B	297	1.15	(1.07, 1.23)	<0.0001	1.12	(1.06, 1.19)	0.0002
C	132	1.12	(1.04, 1.22)	0.0046	1.07	(1.00, 1.15)	0.0445
D	712	1.05	(1.01, 1.10)	0.0201	1.05	(1.01, 1.09)	0.014

Adjusted age, BMI, bFSH, bE2, dose of Gn, type of PCOS, years of infertility, cycle outcome, if not stratified.

Subjects were stratified by PCOS phenotype. For phenotype A, CPR and CLBR increased by 11% and 14%, respectively, with increasing oocyte number [OR = 1.11 (95% CI: 1.03, 1.26) and OR = 1.14 (95% CI: 1.07, 1.21)] (**Figures 3E, F**; **Table 5**). For phenotype B, the CPR had a curve relationship with the number of oocytes. When the number of oocytes retrieved was less than 15, the CPR increased by 22% as the number of oocytes increased, and when the number of oocytes was more than 15, the CPR tended to be stable [OR = 1.22 (95% CI: 1.12, 1.32)]. For phenotype B, the CLBR increased by 12% with increasing oocyte number [OR = 1.12 (95% CI: 1.06, 1.19)] (**Figures 3E, F**; **Tables 5**, **6**). For phenotype C, the CPR had a curve relationship with the number of oocytes. When the number of oocytes retrieved was less than 15, there was no significant correlation between the number of oocytes and the CPR. When the number of oocytes was greater than 15, the CPR increased by 30% with increasing oocyte number [OR = 1.30 (95% CI: 0.17, 1.57)]. For phenotype C, the CLBR increased by 7% with increasing oocyte number [R = 1.07 (95% CI: 1.00, 1.15)] (**Figures 3E, F**; **Tables 5**, **6**). For phenotype D, the number of oocytes retrieved had a curve relationship with the CPR and CLBR. When the number of oocytes retrieved was less than 15, the CPR and CLBR increased by 15% and 14%, respectively, as the number of retrieved oocytes increased [OR = 1.15 (95% CI: 1.07, 1.24) and OR = 1.14 (95% CI: 1.06, 1.22)]; when more than 15, the CPR and CLBR tended to be stable (**Figures 3E, F**; **Table 6**).

**Table 6:** Effect of age, BMI, and type of PCOS on the association between the number of oocytes retrieved and cumulative clinical outcomes.

	No. of oocyte	*N*	The cumulative pregnancy rate				The cumulative live birth rate			
			OR	95% CI	*p*-value	LRT test	OR	95% CI	*p*-value	LRT test
Age <35	<15	644	1.17	(1.12, 1.23)	<0.0001	<0.001	1.16	(1.11, 1.22)	<0.0001	<0.001
	>15	691	1.02	(0.97, 1.06)	0.4697		1.02	(0.98, 1.06)	0.4341	
Age ≥35	<15	51	1.05	(0.90, 1.23)	0.5464	0.852	1.03	(0.88, 1.20)	0.7181	0.444
	>15	33	1.08	(0.85, 1.37)	0.5148		1.14	(0.95, 1.38)	0.1683	
BMI <25	<15	331	1.18	(1.10, 1.26)	<0.0001	0.002	1.15	(1.08,1.23)	<0.0001	0.003
	>15	394	1	(0.95, 1.06)	0.9494		1	(0.95, 1.05)	0.9558	
BMI ≥25	<20	505	1.13	(1.08, 1.19)	<0.0001	0.224	1.14	(1.08, 1.19)	0.7181	0.312
	>20	178	1.04	(0.94, 1.15)	0.4744		1.06	(0.95, 1.18)	0.278	
Type of PCOS										
A	<15	120	1.18	(1.03, 1.34)	0.0166	0.309	1.17	(1.04, 1.31)	0.0079	0.58
	>15	158	1.05	(0.94, 1.18)	0.3756		1.11	(0.99, 1.24)	0.0719	
B	<15	205	1.22	(1.12, 1.32)	<0.0001	0.016	1.17	(1.08, 1.27)	<0.0001	0.086
	>15	92	0.97	(0.87, 1.08)	0.6337		1.01	(0.90, 1.13)	0.8768	
C	<15	56	0.98	(0.83, 1.15)	0.8089	0.043	1.09	(0.95, 1.26)	0.2149	0.757
	>15	76	1.3	(1.07, 1.57)	0.0077		1.06	(0.96, 1.17)	0.2677	
D	<15	314	1.15	(1.07, 1.24)	0.0002	0.003	1.14	(1.06, 1.22)	0.0003	0.007
	>15	398	0.98	(0.93, 1.03)	0.4633		0.99	(0.94, 1.04)	0.6633	

Adjustment factors included age BMI, bFSH, bE2, dose of Gn, type of PCOS, years of infertility, cycle outcome, and if not stratified.

LRT test, Logarithmic likelihood ratio test.

**Figure 4:**
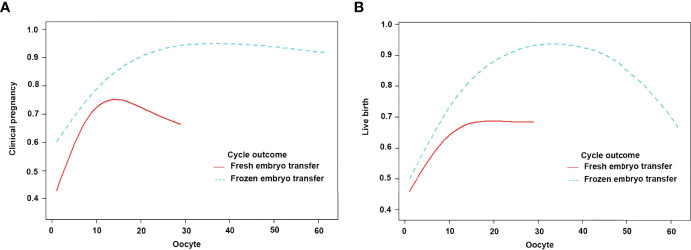
Patients were classified according to fresh/frozen embryo transfer stratification, curve fitting between the number of oocytes retrieved, and clinical outcomes. The adjusted smoothed plots between the number of oocytes retrieved with the clinical pregnancy rate and the live birth rate were based on a two-piecewise regression model **(A, B)**. The red line represents fresh embryo transfer, and the blue line represents frozen embryo transfer. Adjustment factors included age, BMI, bFSH, bE2, dose of Gn, type of PCOS, and years of infertility.

The subjects were stratified according to the transplanted fresh embryo/frozen embryo. When fresh embryos were transplanted when the number of oocytes was less than 11, the clinical pregnancy rate increased by 17%, and the live birth rate increased by 12% with each increase in the number of oocytes. After more than 11, the clinical pregnancy rate and live birth rate plateaued. When frozen embryos were transplanted when the number of oocytes was less than 25, the clinical pregnancy rate and the live birth rate increased by 11% and 13% with each increase in the number of oocytes. After more than 25, the live birth rate plateaued, but the clinical pregnancy rate decreased by 8% with each increase in the number of oocytes (**Figure 4**; **Table 7**).

**Table 7:** Effect of fresh/frozen embryo transfer on the association between the number of oocytes retrieved and clinical outcomes.

No. of oocyte	*N*	The clinical pregnancy rate				The live birth rate			
		OR	95% CI	*p*-value	LRT test	OR	95% CI	*p*-value	LRT test
Fresh embryo transfer									
<11	371	1.17	(1.08,1.26)	<0.0001	0.001	1.12	(1.06,1.19)	0.0001	0.047
>11	488	0.97	(0.92,1.02)	0.2631		0.98	(0.91,1.05)	0.5845	
Frozen embryo transfer									
<25	368	1.11	(1.06,1.17)	<0.0001	0.029	1.13	(1.07,1.19)	<0.0001	0.003
>25	192	0.92	(0.86,0.99)	0.0249		0.96	(0.91,1.02)	0.2197	

Adjustment factors included age, BMI, bFSH, bE2, dose of Gn, type of PCOS, and years of infertility.

LRT test, Logarithmic likelihood ratio test.

### Pregnancy and Live Birth After Fresh Embryo Transfer

**Figure 5:**
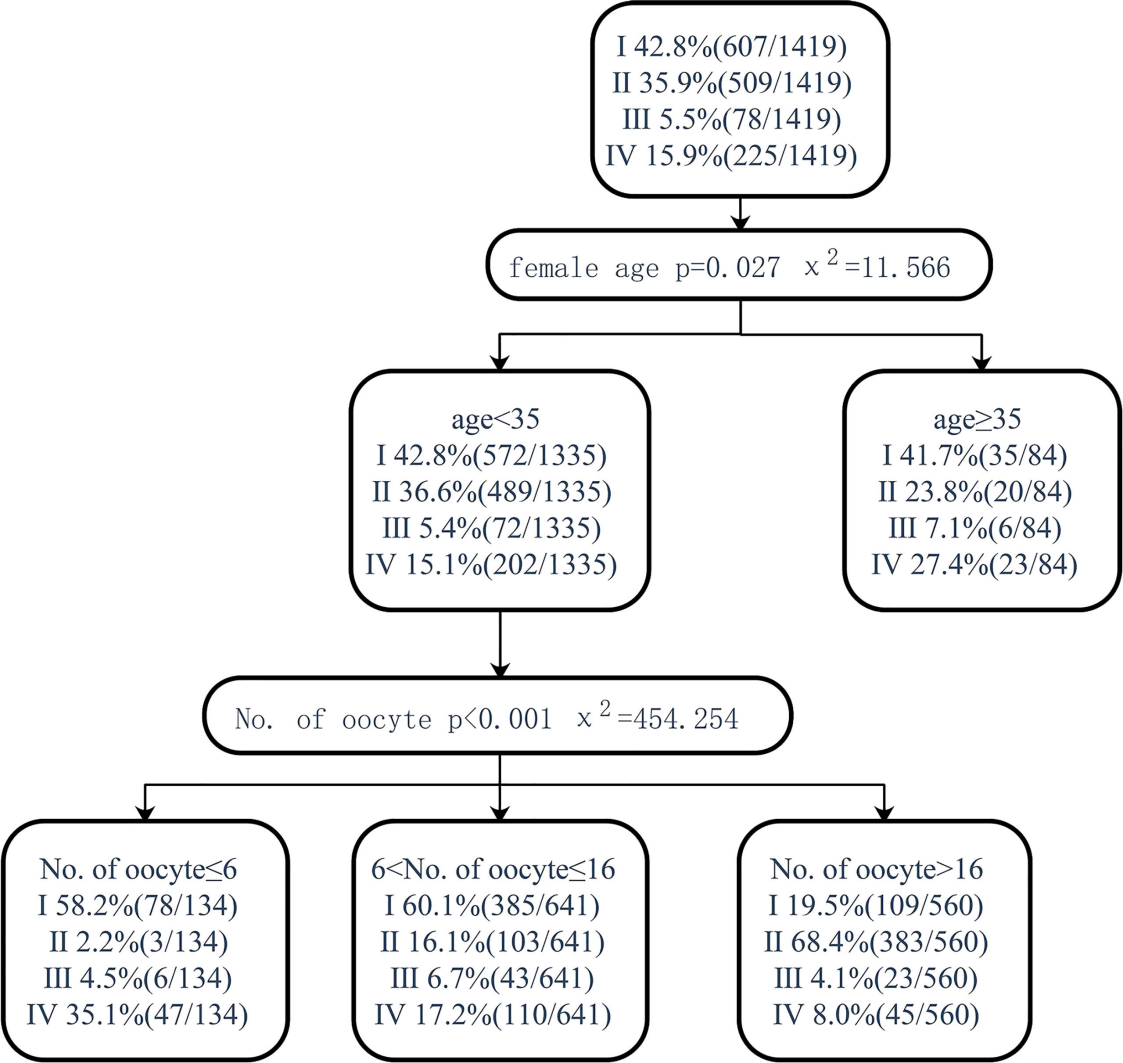
Decision tree on pregnancy after fresh embryo transfer. I, clinical pregnancy after fresh embryo transfer; II, clinical pregnancy after the first transfer using frozen embryo; III, clinical pregnancy after multiple embryo transfers; IV, no clinical pregnancy after embryo exhaustion.

**Figure 6:**
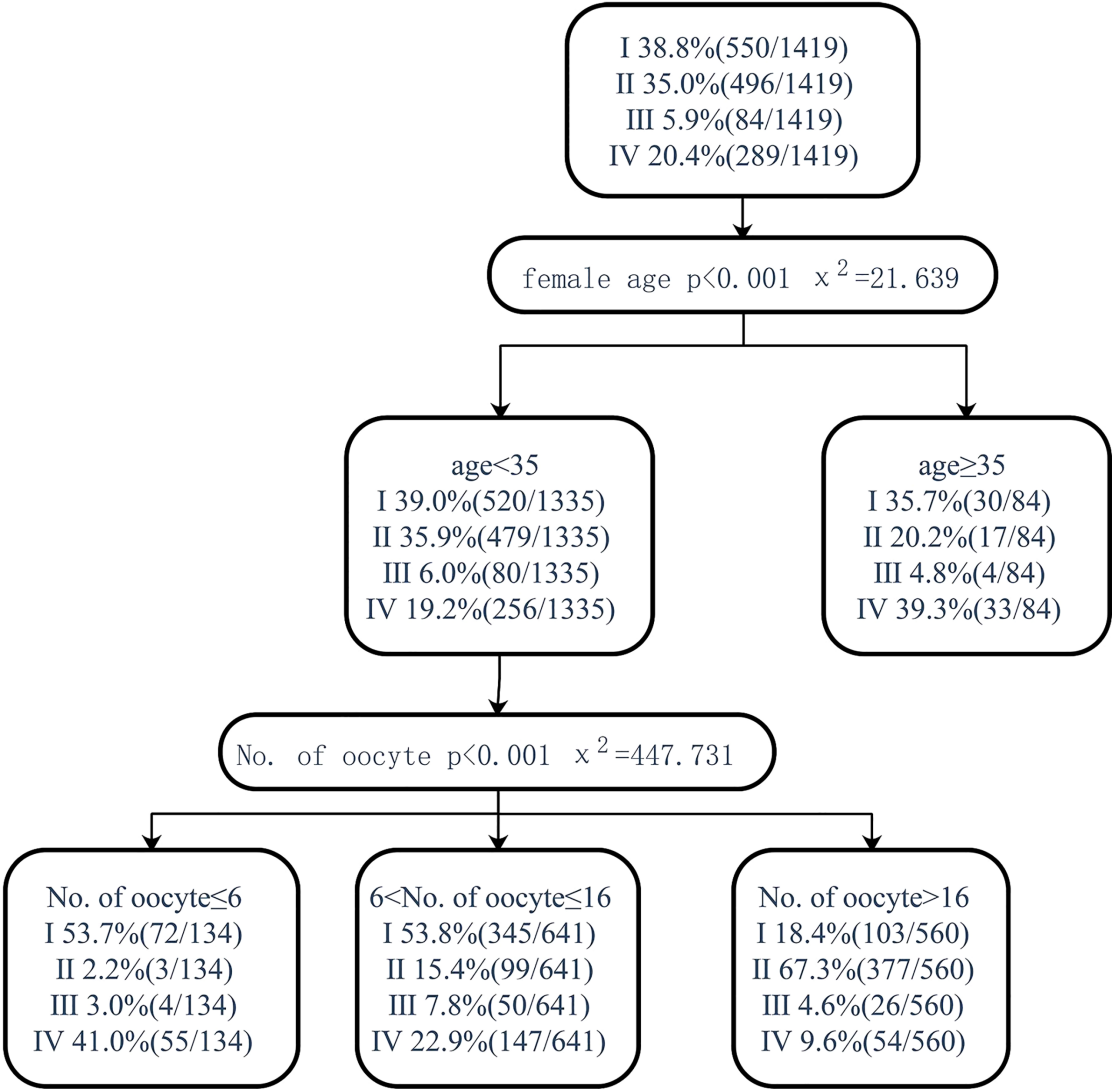
Decision tree on live birth after fresh embryo transfer. I, live birth after fresh embryo transfer; II, live birth after the first transfer using frozen embryo; III, live birth after multiple embryo transfers; IV, no live birth after embryo exhaustion.

Pregnancy after fresh embryo transfer and live birth after fresh embryo transfer were analyzed through the classification decision tree. For patients younger than 35 years old, those with less than or equal to 6 oocytes and those with 7–16 oocytes had a similar proportion of pregnancy and live births with fresh ET but higher than 16 oocytes (58.2% vs. 60.1% vs. 19.5%, 53.7 vs. 53.8% vs. 18.4%). Patients younger than 35 years old had a higher proportion of pregnancy and live births (58.2% vs 42.8%, 53.7% vs 38.8%) of fresh ET in those with less than or equal to 6 oocytes retrieved, but the proportion of no live birth after embryo exhaustion was higher than the total patients (35.1% vs. 15.9%, 41.0% vs. 20.4%). When the age was over 35, though there was no significant correlation with the number of oocytes retrieved, the proportion of patients who could not become pregnant or deliver alive after using all embryos was significantly higher than that of patients less than 35 years old (27.4% vs. 15.1%, 39.3% vs. 19.2%) (**Figures 5**, **6**).

## Discussion

In our study, the number of oocytes retrieved in PCOS patients was closely related to the CPR and CLBR. The CPR and CLBR of PCOS patients reached their peak when the number of oocytes retrieved was 15, and then stabilized. Similarly, a study showed that PCOS patients with 10–15 oocytes could get a CLBR of 81.91%. After that, even if the number of oocytes retrieved increased, the CLBR did not change significantly (7).

The possible reason is that the number of euploid embryos in PCOS patients increases with the number of retrieved oocytes, and the increase in the number of available embryos increases the chance of live births in PCOS patients (12, 13). PCOS patients increase the number of euploid embryos by increasing the number of oocytes obtained, thereby increasing live births. Therefore, we should also pay attention to the quality of oocytes in PCOS patients rather than simply increasing the number of oocytes.

Age is an important factor affecting female fertility, and the number of oocytes retrieved at different ages may have an impact on the CLBR. Patients aged 18–35 were recommended to retrieve up to 25 oocytes and 30 oocytes in women between 36 and 44 years old (14). Quite different from that, our result remains stable in patients younger than 35 years of age, but there was no correlation in PCOS patients older than 35 years. Differences in the study populations could have contributed to the differences in the results among the various studies. PCOS patients have a reproductive advantage over non-PCOS patients. Compared with non-PCOS patients, the number of oocytes obtained in PCOS patients and the CLBR decline with age are slower (15). Therefore, under the same age, PCOS patients could reach a maximum CLBR with a small number of oocytes retrieved. In this study, the small sample size of PCOS patients older than 35 years may be the main reason for the lack of correlation between the number of oocytes and the CLBR.

When the patient’s age was between 35 and 40 years old, there was no significant correlation between cumulative pregnancy and cumulative live birth and the number of oocytes. However, the proportion of patients who have not delivered a live birth after using all their embryos was significantly higher than that of patients younger than 35 years. However, studies have shown that the number of oocyte retrievals in women under the age of 41 is significantly associated with CLBR (16). Retrieving 10–14 oocytes in women aged 35–40 years can achieve high CLBR while maintaining acceptably low OHSS rates (17). In addition, another study showed that women aged 35–44 with more than 30 oocytes can achieve high CPR (14). However, it is more difficult for patients over 35 to obtain more than 30 oocytes. PCOS patients were maintained with IVF until age 38, after which pregnancy rates declined, although the number of oocytes obtained with IVF remained stable (18). The sample size should be expanded for further study.

In this study, PCOS patients without overweight or obesity achieved the best CPR and CLBR when the number of oocytes obtained reached 15. CPR and CLBR can be increased by increasing the number of oocytes retrieved, but at a lower rate than in patients without overweight or obesity. Overweight and obesity are independent risk factors for cumulative live birth rates and increase the risk of miscarriage (19), and overweight and obesity occur more frequently in PCOS patients than in patients without PCOS, which affects the fertility of PCOS patients. On the one hand, obesity affects the function of the endometrium in PCOS patients through inflammation, oxidative stress, and other pathways that impair the decidual formation and hinder embryo implantation (20). On the other hand, obesity can impair oocyte function through abnormal spindle and chromosome pairing or by affecting the physiological microenvironment in the follicular fluid (21). For overweight and obese patients, weight control and normal glucose and lipid metabolism are even more important than increasing the number of oocytes retrieved.

Patients with four PCOS phenotypes can increase CPR and CLBR by increasing the number of oocytes retrieved. Only the patients with the D phenotype have a trend in the relationship between the number of oocytes retrieved and CLBR when the number of retrieved oocytes reaches 15 in stability. In addition, patients with the C phenotype had a lower percentage of CLBR by increasing the number of oocytes retrieved. Studies have shown that patients with a hyperandrogenic phenotype (A and C phenotypes) have a significantly lower CLBR than patients with a nonhyperandrogenic phenotype (D phenotype) (22). Oocyte function in PCOS patients is associated with the PCOS phenotype where hyperandrogenemia leads to premature luteinization of granulosa cells and premature oocyte maturation by affecting the feedback of ovarian steroids to the HPO axis, and hyperandrogenemia also alters follicles’ liquid microenvironment (21). High testosterone levels affect the metabolism of glucose in the endometrium of PCOS patients, causing local insulin resistance, resulting in impaired endometrial function (23). Patients with the D phenotype, as the phenotype with the least symptoms, have no effect of hyperandrogenism, and the best CLBR can be obtained when the number of oocytes retrieved reaches 15. Therefore, more attention should be paid to patients with A and B phenotypes, especially C phenotypes during assisted reproduction.

Our study showed that, among PCOS patients with fresh ET, 11 oocytes could achieve the best clinical pregnancy rate and live birth rate. Among PCOS patients with their first FET after whole embryo freezing, 25 oocytes could achieve the best clinical pregnancy rate and live birth rate. Later, as the number of oocytes harvested increases, the live birth rate for the first transplant gradually decreases. A study showed that 9 oocytes could optimize the chance of live birth following fresh transfer in women with normal ovarian reserve (24). Retrieving at least 20 oocytes may be the optimal cutoff point for FET in order to maximize the likelihood of a successful pregnancy and live birth while minimizing complications (25). Studies have shown that women with PCOS have an increased risk of miscarriage, especially during fresh ET cycles, compared to women without PCOS (26, 27). Therefore, patients with PCOS, especially during fresh ET, need more oocytes to increase their chances of live birth by increasing the number of available embryos and high-quality embryos.

Our study also showed that the number of oocytes retrieved between 7 and 16 could obtain a high proportion of fresh ET pregnancy and live birth in patients younger than 35 years old. Patients receiving IVF hope to obtain clinical pregnancy and a live birth in the shortest time. In women with PCOS, FET appears to be superior to fresh ET for women with more than 16 oocytes (9). With the permission of multiple children in a family in China, many patients who give birth to their first child through assisted reproduction were ready to receive children who have undergone FET. In this case, assisted reproduction centers should help patients get pregnant quickly and efficiently (28). If the number of oocytes retrieved was too high due to the use of radical ovarian stimulation protocol, our assisted reproductive center would choose whole embryo freezing to avoid transfer failure and complications such as OHSS, and to reduce the chance of fresh ET. Although the proportion of live births from fresh embryo transplantation increased in patients with less than or equal to 6 oocytes, the proportion of patients who were unable to live birth even after using up all embryos also increased. Patients with a small number of oocytes are at risk of embryo freeze–thaw failure by poor embryo quality, so they tend to choose fresh ET. At the same time, in a population with a small number of oocytes, the possibility of live birth may be reduced due to embryo quality and maternal factors. Controlling the number of oocytes to 7–16 can obtain a high proportion of fresh ET pregnancy and even a live birth rate, which reduces the time of treatment and saves medical costs.

However, it is not that the more oocytes retrieved, the better the prognosis of the patient. Retrieving more than 15 oocytes in a fresh ET cycle significantly increases the risk of OHSS but not the LB rate (29). When the number of oocytes exceeds 20, excess oocytes do not imply higher CLBR but higher residual embryo rates, higher doses of gonadotropins, and longer days of ovarian stimulation, and only less than half of the embryos are transferred and the rest are either frozen indefinitely or abandoned, which is a huge waste (7). On the one hand, a mild ovarian stimulation protocol should be selected to obtain an appropriate number of oocytes and, on the other hand, to increase the quality of oocytes and embryos and to obtain a higher CLBR.

Compared with the assessment of pregnancy outcomes of fresh embryo transfer only, our study used first transfer outcomes, including fresh embryo transfer outcomes and first frozen embryo transfer outcomes, for a more adequate assessment. This study has limitations. The relatively small number of patients over the age of 35 and the C phenotype may mask differences in the data. This study lacks research on the relationship between the incidence of OHSS, thrombosis, and other complications and the number of oocytes retrieved. In this study, PCOS patients only received GnRH-ant protocol, and there was a lack of comparison between different ovulation stimulation protocols. This study is a clinical retrospective study, and the limitations of time and data can easily lead to biased results. A multicenter large-sample study is needed to further confirm the conclusions of this study.

## Conclusion

In PCOS patients, the best CLBR is obtained with 15 oocytes, but 7–16 oocytes can be obtained in the shortest time, also known as fresh ET live birth. In clinical practice, general conditions such as age, personal preference, and treatment benefits of PCOS patients should be fully evaluated, and an appropriate ovarian stimulation program should be selected to carefully control the balance between the number of oocytes retrieved and live birth.

## Data Availability Statement

The data analyzed in this study is subject to the following licenses/restrictions: The datasets generated and/or analyzed during the current study are available from the corresponding author on reasonable request. Requests to access these datasets should be directed to ZZ (doctor_zhaozhao@sina.com).

## Ethics Statement

The studies involving human participants were reviewed and approved by the research ethics committee of the Second Hospital of Hebei Medical University. Written informed consent for participation was not required for this study in accordance with the national legislation and the institutional requirements.

## Author Contributions

RJ: acquisition, analysis and interpretation of data and writing manuscript. YL and RJ: analysis and interpretation of data. XZ, LZ, PC, and MC: acquisition of data. ZZ: substantial contribution in design and conception of the study. All authors read and approved the final manuscript.

## Funding

This study was supported by the Natural Science Foundation of Hebei Province (Beijing-Tianjin-Hebei Cooperation Special Project) (H2019206712).

## Conflict of Interest

The authors declare that the research was conducted in the absence of any commercial or financial relationships that could be construed as a potential conflict of interest.

## Publisher’s Note

All claims expressed in this article are solely those of the authors and do not necessarily represent those of their affiliated organizations, or those of the publisher, the editors and the reviewers. Any product that may be evaluated in this article, or claim that may be made by its manufacturer, is not guaranteed or endorsed by the publisher.

## References

[1] Magnusson A Kallen K Thurin-Kjellberg A Bergh C. The Number of Oocytes Retrieved During Ivf: A Balance Between Efficacy and Safety. Hum Reprod (2018) 33(1):58–64. doi: 10.1093/humrep/dex334 29136154

[2] Drakopoulos P Blockeel C Stoop D Camus M de Vos M Tournaye H. Conventional Ovarian Stimulation and Single Embryo Transfer for Ivf/Icsi. How Many Oocytes Do We Need to Maximize Cumulative Live Birth Rates After Utilization of All Fresh and Frozen Embryos? Hum Reprod (2016) 31(2):370–6. doi: 10.1093/humrep/dev316 26724797

[3] Polyzos NP Drakopoulos P Parra J Pellicer A Santos-Ribeiro S Tournaye H. Cumulative Live Birth Rates According to the Number of Oocytes Retrieved After the First Ovarian Stimulation for *In Vitro* Fertilization/Intracytoplasmic Sperm Injection: A Multicenter Multinational Analysis Including Approximately 15,000 Women. Fertil Steril (2018) 110(4):661–70 e1. doi: 10.1016/j.fertnstert.2018.04.039 30196963

[4] Law YJ Zhang N Kolibianakis EM Costello MF Keller E Chambers GM. Is There an Optimal Number of Oocytes Retrieved at Which Live Birth Rates or Cumulative Live Birth Rates Per Aspiration Are Maximized After Art? A Systematic Review. Reprod BioMed Online (2021) 42(1):83–104. doi: 10.1016/j.rbmo.2020.10.008 33390313

[5] Doherty LF Martin JR Kayisli U Sakkas D Patrizio P. Fresh Transfer Outcome Predicts the Success of a Subsequent Frozen Transfer Utilizing Blastocysts of the Same Cohort. Reprod BioMed Online (2014) 28(2):204–8. doi: 10.1016/j.rbmo.2013.09.030 24365019

[6] Bhide P Pundir J Gudi A Shah A Homburg R Acharya G. The Effect of Myo-Inositol/Di-Chiro-Inositol on Markers of Ovarian Reserve in Women With Pcos Undergoing Ivf/Icsi: A Systematic Review and Meta-Analysis. Acta Obstet Gynecol Scand (2019) 98(10):1235–44. doi: 10.1111/aogs.13625 30993683

[7] Chen YH Wang Q Zhang YN Han X Li DH Zhang CL. Cumulative Live Birth and Surplus Embryo Incidence After Frozen-Thaw Cycles in Pcos: How Many Oocytes Do We Need? J Assist Reprod Genet (2017) 34(9):1153–9. doi: 10.1007/s10815-017-0959-6 PMC5581780 28580513

[8] Qiao J Feng HL. Extra- and Intra-Ovarian Factors in Polycystic Ovary Syndrome: Impact on Oocyte Maturation and Embryo Developmental Competence. Hum Reprod Update (2011) 17(1):17–33. doi: 10.1093/humupd/dmq032 20639519 PMC3001338

[9] Wei D Yu Y Sun M Shi Y Sun Y Deng X. The Effect of Supraphysiological Estradiol on Pregnancy Outcomes Differs Between Women With Pcos and Ovulatory Women. J Clin Endocrinol Metab (2018) 103(7):2735–42. doi: 10.1210/jc.2018-00613 29718297

[10] Alpha Scientists in Reproductive M Embryology ESIGo. The Istanbul Consensus Workshop on Embryo Assessment: Proceedings of an Expert Meeting. Hum Reprod (2011) 26(6):1270–83. doi: 10.1093/humrep/der037 21502182

[11] Embryology ESIGo Alpha Scientists in Reproductive M. The Vienna Consensus: Report of an Expert Meeting on the Development of Art Laboratory Performance Indicators. Hum Reprod Open (2017) 2017(2):hox011. doi: 10.1093/hropen/hox011 31486806 PMC6276649

[12] Labarta E Bosch E Mercader A Alama P Mateu E Pellicer A. A Higher Ovarian Response After Stimulation for Ivf Is Related to a Higher Number of Euploid Embryos. BioMed Res Int (2017) 2017:5637923. doi: 10.1155/2017/5637923 28428962 PMC5385900

[13] Weghofer A Munne S Chen S Barad D Gleicher N. Lack of Association Between Polycystic Ovary Syndrome and Embryonic Aneuploidy. Fertil Steril (2007) 88(4):900–5. doi: 10.1016/j.fertnstert.2006.12.018 17433813

[14] Law YJ Zhang N Venetis CA Chambers GM Harris K. The Number of Oocytes Associated With Maximum Cumulative Live Birth Rates Per Aspiration Depends on Female Age: A Population Study of 221 221 Treatment Cycles. Hum Reprod (2019) 34(9):1778–87. doi: 10.1093/humrep/dez100 31398253

[15] Li J Liu X Hu L Zhang F Wang F Kong H. A Slower Age-Related Decline in Treatment Outcomes After the First Ovarian Stimulation for *in Vitro* Fertilization in Women With Polycystic Ovary Syndrome. Front Endocrinol (Lausanne) (2019) 10:834. doi: 10.3389/fendo.2019.00834 31866942 PMC6906164

[16] Devesa M Tur R Rodriguez I Coroleu B Martinez F Polyzos NP. Cumulative Live Birth Rates and Number of Oocytes Retrieved in Women of Advanced Age. A Single Centre Analysis Including 4500 Women >/=38 Years Old. Hum Reprod (2018) 33(11):2010–7. doi: 10.1093/humrep/dey295 30272168

[17] Zhou J Wang B Hu Y Sun H. Association Between the Number of Oocytes Retrieved and Cumulative Live Birth Rate in Women Aged 35-40 Years Undergoing Long Gnrh Agonist Ivf/Icsi Cycles. Arch Gynecol Obstet (2017) 296(5):1005–12. doi: 10.1007/s00404-017-4503-9 28879481

[18] Hwang YI Cha SW Song IO Yang KM Min EG Kim HO. Fertility of Patients With Polycystic Ovary Syndrome Undergoing *In Vitro* Fertilization by Age. Int J Gynaecol Obstet (2016) 135(1):91–5. doi: 10.1016/j.ijgo.2016.03.033 27406030

[19] Ding W Zhang FL Liu XC Hu LL Dai SJ Li G. Impact of Female Obesity on Cumulative Live Birth Rates in the First Complete Ovarian Stimulation Cycle. Front Endocrinol (Lausanne) (2019) 10:516. doi: 10.3389/fendo.2019.00516 31428050 PMC6687867

[20] Palomba S Piltonen TT Giudice LC. Endometrial Function in Women With Polycystic Ovary Syndrome: A Comprehensive Review. Hum Reprod Update (2021) 27(3):584–618. doi: 10.1093/humupd/dmaa051 33302299

[21] Palomba S Daolio J La Sala GB. Oocyte Competence in Women With Polycystic Ovary Syndrome. Trends Endocrinol Metabol: TEM (2017) 28(3):186–98. doi: 10.1016/j.tem.2016.11.008 27988256

[22] De Vos M Pareyn S Drakopoulos P Raimundo JM Anckaert E Santos-Ribeiro S. Cumulative Live Birth Rates After Ivf in Patients With Polycystic Ovaries: Phenotype Matters. Reprod BioMed Online (2018) 37(2):163–71. doi: 10.1016/j.rbmo.2018.05.003 29778554

[23] Palomba S. Is Fertility Reduced in Ovulatory Women With Polycystic Ovary Syndrome? An Opinion Paper. Hum Reprod (2021) 36(9):2421–8. doi: 10.1093/humrep/deab181 34333641

[24] Datta AK Campbell S Felix N Singh JSH Nargund G. Oocyte or Embryo Number Needed to Optimize Live Birth and Cumulative Live Birth Rates in Mild Stimulation Ivf Cycles. Reprod BioMed Online (2021) 43(2):223–32. doi: 10.1016/j.rbmo.2021.02.010 34140227

[25] Xu B He YQ Wang Y Lu Y Hong Y Wang Y. Frozen Embryo Transfer or Fresh Embryo Transfer: Clinical Outcomes Depend on the Number of Oocytes Retrieved. Eur J Obstet Gynecol Reprod Biol (2017) 215:50–4. doi: 10.1016/j.ejogrb.2017.05.023 28600920

[26] Sun YF Zhang J Xu YM Cao ZY Wang YZ Hao GM. High Bmi and Insulin Resistance Are Risk Factors for Spontaneous Abortion in Patients With Polycystic Ovary Syndrome Undergoing Assisted Reproductive Treatment: A Systematic Review and Meta-Analysis. Front Endocrinol (Lausanne) (2020) 11:592495. doi: 10.3389/fendo.2020.592495 33343510 PMC7744738

[27] Li HW Lee VC Lau EY Yeung WS Ho PC Ng EH. Cumulative Live-Birth Rate in Women With Polycystic Ovary Syndrome or Isolated Polycystic Ovaries Undergoing *in-Vitro* Fertilisation Treatment. J Assist Reprod Genet (2014) 31(2):205–11. doi: 10.1007/s10815-013-0151-6 PMC3933606 24337962

[28] Zhu XL Zhao ZM Du YJ Zhou L Wang Y Sun QY. The Optimal Number of Embryo Cells for Effective Pregnancy and Decrease of Multiple Pregnancy Rate in Frozen-Thawed Embryo Transfer. Hum Cell (2021) 34(3):836–46. doi: 10.1007/s13577-021-00516-0 33689158

[29] Steward RG Lan L Shah AA Yeh JS Price TM Goldfarb JM. Oocyte Number as a Predictor for Ovarian Hyperstimulation Syndrome and Live Birth: An Analysis of 256,381 in Vitro Fertilization Cycles. Fertil Steril (2014) 101(4):967–73. doi: 10.1016/j.fertnstert.2013.12.026 24462057

